# Amino acid transporters as tetraspanin TM4SF5 binding partners

**DOI:** 10.1038/s12276-019-0363-7

**Published:** 2020-01-20

**Authors:** Jae Woo Jung, Ji Eon Kim, Eunmi Kim, Jung Weon Lee

**Affiliations:** 1 Interdisciplinary Program in Genetic Engineering, Seoul National University, Seoul, 08826 Republic of Korea; 2 Department of Pharmacy, Research Institute of Pharmaceutical Sciences, College of Pharmacy, Seoul National University, Seoul, 08826 Republic of Korea

**Keywords:** Metabolomics, Diagnostic markers, Nutrient signalling, Non-alcoholic fatty liver disease

## Abstract

Transmembrane 4 L6 family member 5 (TM4SF5) is a tetraspanin that has four transmembrane domains and can be *N*-glycosylated and palmitoylated. These posttranslational modifications of TM4SF5 enable homophilic or heterophilic binding to diverse membrane proteins and receptors, including growth factor receptors, integrins, and tetraspanins. As a member of the tetraspanin family, TM4SF5 promotes protein-protein complexes for the spatiotemporal regulation of the expression, stability, binding, and signaling activity of its binding partners. Chronic diseases such as liver diseases involve bidirectional communication between extracellular and intracellular spaces, resulting in immune-related metabolic effects during the development of pathological phenotypes. It has recently been shown that, during the development of fibrosis and cancer, TM4SF5 forms protein-protein complexes with amino acid transporters, which can lead to the regulation of cystine uptake from the extracellular space to the cytosol and arginine export from the lysosomal lumen to the cytosol. Furthermore, using proteomic analyses, we found that diverse amino acid transporters were precipitated with TM4SF5, although these binding partners need to be confirmed by other approaches and in functionally relevant studies. This review discusses the scope of the pathological relevance of TM4SF5 and its binding to certain amino acid transporters.

## Cell biology: troubles with trafficking

Interactions between a membrane protein and transporters that move amino acids in and out of cells may play a prominent role in various diseases. The transmembrane protein TM4SF5 is known to regulate the activity of diverse signaling pathways, and has been linked to various liver diseases. Jung Weon Lee and colleagues at Seoul National University review the recently identified interplay between TM4SF5 and transporter proteins that regulate trafficking of the amino acids cysteine and arginine. Through its interactions with cysteine transporters, TM4SF5 appears to contribute to hormetic production of damaging chemicals that can cause the tissue scarring underlying idiopathic pulmonary fibrosis. Evidence also suggests that TM4SF5-facilitated arginine trafficking may contribute to liver cancer recurrence. This protein may also interact with other prominent transporters, and the authors conclude that TM4SF5 may affect other, still undefined pathological processes.

## Introduction

Importing and exporting biological matter in and out of a cell are fundamental and integral processes for homeostasis and survival. In the same context, membrane transporters are core proteins that govern cell status and fate, and it is not surprising that ~10% of the genome encodes transporter and transporter-related genes^1^. Solute carrier transporter families (SLCs) are the second largest family of membrane proteins, consisting of 52 families with almost 400 transporters. SLCs are composed of various transporters with different modes of transport, including ion couplers, exchangers, and passive transporters. SLCs are ubiquitously expressed in almost all human cells, and their intracellular localizations are found on the plasma membrane and the mitochondria, lysosome, and other intracellular organelles. SLCs transport small molecules, and inorganic and organic ions^2,3^, such as sugars (SLC2, 5, 45, 50, and 60)^4^, amino acids (SLC1, 3, 7, 17, 32, 36, 38, and 43)^5–9^, fatty acids (SLC27)^10^, calcium (SLC8, 24, and 64)^11–13^, zinc (SLC30 and 39)^14,15^, and many others. The lists and functions of SLCs are well summarized on the Bioparadigms webpage (https://www.bioparadigms.org/slc/intro.htm). The topology and structure vary among transporters, which are generally composed of a number of hydrophobic helices connected with loops. Some SLCs exist as heterodimers (SLC3A2 with SLC7A5-8, 10–11), and others exist as monomers.

## Amino acid transporters in disease

SLCs transport molecules fundamental for homeostatic survival and growth such that mutations and either enhancing or attenuating malfunctions of SLCs can cause diseases. Approximately 20% of human SLC mutations are related to diseases, according to the Online Mendelian Inheritance in Man (OMIM) database^16^.

### Amino acid transporters and inflammatory diseases

Inflammation is a normal response to injury in the human body. This response requires immune cells to react to cellular damage, and this reaction includes infiltration of and metabolic changes in immune cells and leads to the modification of cell fate and effector function. An arginine transporter, SLC7A2, was shown to play a pivotal role in inflammation-associated colon tumorigenesis^17^; the *Slc7a2*^*−/−*^ mice serving as colon cancer models were more susceptible to azoxymethane-dextran sulfate sodium-induced switching of macrophages to the M2 phenotype and activation. Levels of SLC15A1, a peptide transporter, were also increased by pro-inflammatory cytokines in conjunction with increases in tumor necrosis factor-α and interferon-γ, being involved in inflammatory bowel disease (IBD)^18^. SLC22A genes were also shown to be high susceptibility genes for IBD and to be located in a genetic location called IBD locus 5^19^.

### Amino acid transporters and liver diseases

Because the liver is a central organ of metabolic homeostasis, malfunction(s) to the mechanism of external nutrient import into liver cells can lead to serious pathological diseases. Liver disease is chronic and develops in an inflammatory environment. Non-alcoholic fatty liver disease (NAFLD) is characterized by excessive lipid accumulation in the liver and is often closely related to obesity. Because excessive glucose can be stored in the form of lipids, it is logically related glucose transporters in NAFLD. SLC2A1 (GLUT1), a high-affinity glucose transporter expressed ubiquitously, has been shown to be a susceptibility factor of NAFLD^20^; several single nucleotide polymorphisms (SNPs) are also closely related to NAFLD but not to type 2 diabetes. Trehalose, a SLC2 family inhibitor also known as a sugar inhibitor, has been shown to dramatically ameliorate NAFLD symptoms in mouse models by mimicking starvation to induce autophagy^21^. Genetically ablating *Slc7a3* in zebrafish, in which it encodes a cationic amino acid transporter, results in defects in arginine-dependent nitric oxide synthesis, which leads to hepatic steatosis^22^.

### Amino acid transporters and fibrosis

Chronic tissue injury can lead to the accumulation of a diverse extracellular matrix (ECM) in an inflammatory environment. Chronic injury of epithelial cells can lead to transformation to mesenchymal cells and/or activation of myofibroblasts to promote ECM production and deposition^23^. Although diverse ECMs are deposited in tissue or organs, most studies on fibrosis have focused on the collagen network, which accounts for 30% of all proteins in the organisms^24^. Collagens are generally produced by activated myofibroblasts^24^. Emerging consensus on the sources of fibrogenic cells provides the rationale and opportunity for investigating increasingly diverse ECM proteins in addition to collagens and different cell types, including epithelial cells, in addition to fibroblasts. We recently reported that activated myofibroblasts promote collagen expression, whereas epithelial cells induce laminin expression, in vitro, upon activation of the TGFβ1 signaling pathway and chemical induction of fibrosis in animal tissues^25,26^.

In addition, certain amino acid transporters have been shown to be involved in fibrosis. Analysis of allergic airway inflammation and bleomycin-induced inflammation in CAT2 (cationic amino acid transporter 2) deficient mice has shown that, although inflammation is independent of CAT2 expression, bleomycin-induced fibrosis is dependent on CAT2^27^. We have recently reported that a cystine/glutamate antiporter, the xc^−^ system, is involved in the regulation of intracellular glutathione levels and reactive oxygen species (ROS) levels during TM4SF5-mediated pulmonary fibrosis^28^ (see the below section for details).

### Amino acid transporters and cancer

Cancer cells have some functions, such as continuous growth and proliferation, that require large amounts of energy; therefore, cancer cells undergo metabolic change that results in rapid energy production. Sufficient energy production is an important requirement for the survival and rapid growth of cancer cells. Glucose is a major energy source for cancer cells, and amino acids, lipids, and other nutrients are imported from the extracellular environment by many types of transporters found in the plasma membrane. The SLC family mainly transports nutrients with high affinity and specificity, but abnormal expression or regulation of SLCs can lead to poor prognoses of many cancers. The regulation of SLC expression and activation could therefore be a rate-limiting factor for tumor progression. The glucose-transporting proteins (GLUTs) in the SLC family are important in cancer development because of the Warburg effect. Otto Warburg described the fate of glucose transported into cancer cells, indicating that cancer cells tend to undergo glycolysis, even in the presence of oxygen, instead of oxidative phosphorylation^29^. Glycolysis is less efficient in terms of generating ATP (but faster), requiring cancer cells to make more glucose to sustain a high rate of proliferation. Cancer cells require not only large amounts of glucose for energy production but also amino acids, which are the carbon sources for the synthesis of biomolecules necessary for cancer cell growth and survival. In addition to the SLC2 family, the SLC7 family of cationic amino acid transporters is overexpressed in many cancer types. SLC7A5, which is upregulated by hypoxia-inducible factor 2a, and SLC7A11 (a cystine/glutamate exchanger) have been found to be highly expressed in many cancer types^30,31^. Different types of amino acid transporters are also upregulated in cancer tissues: SLC1A5 (sodium-dependent neutral amino acid transporter type 2, which transports glutamine, asparagine, alanine, serine, and cysteine)^32^, SLC7A5 [LAT1 (large neutral amino acid transporter small subunit 1), which transports phenylalanine, tyrosine, leucine, histidine, methionine, and tryptophan]^33^, and SLC6A14 (sodium-dependent and chloride-dependent transporter, which mediates neutral and cationic amino acid uptake)^34^.

## TM4SF5 and amino acid transporters

### Tetraspanin TM4SF5

**Fig. 1:**
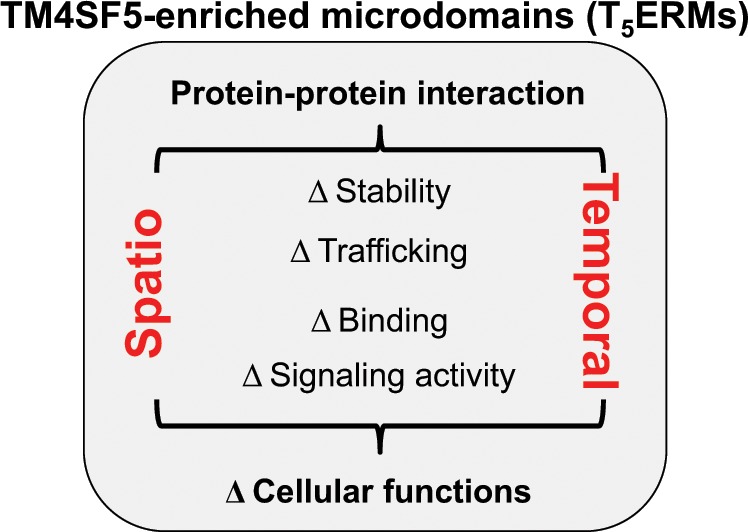
Biological roles of TM4SF5-enriched microdomains (T_5_ERMs) on membranes. Within the T_5_ERMs, TM4SF5 can form massive protein-protein complexes with amino acid transporters, growth factor receptors, integrins, and other tetraspanins. TM4SF5 can thereby affect the expression/stability, trafficking/translocation, binding, and/or signaling activity of binding partners in a spatiotemporal manner, leading to changes in cellular functions during homeostasis or pathological diseases.

TM4SF5 is a membrane protein and is a member of the tetraspanin family, with four transmembrane domains, a cytosolic N-terminus and C-terminus, and an intracellular loop. It undergoes *N*-glycosylation at the N138 and N155 residues and palmitoylation at the cysteine residues near the cytosolic boundary of the transmembrane domains^35^. Similar to other tetraspanins, TM4SF5 has been shown to interact with diverse membrane proteins and receptors on membranes, resulting in TM4SF5-enriched microdomains (i.e., T_5_ERMs). TM4SF5 may regulate the expression, stability, binding, and/or signaling activity of binding partners in T_5_ERMs in a spatiotemporal manner (Fig. 1).

TM4SF5 is overexpressed in different cancer types, including liver cancer^36^. In addition, CCl_4_-induced liver fibrosis^37^ and bleomycin-induced lung fibrosis^28^ involve TM4SF5 expression, and the chemically induced fibrotic phenotypes in animal models are blocked by the specific TM4SF5 small inhibitory compound 4'-(*p*-toluenesulfonylamido)-4-hydroxychalcone (TSAHC)^38^. The relationship between TM4SF5 and SLCs can be additionally observed through the results from coexpression analyses of The Cancer Genome Atlas (TCGA) database.

TM4SF5 has been shown to form a protein-protein complex on the cell surface with CD151^39^, EGFR, and integrin α5^40^ to play promigratory roles; with CD44^41^ and CD133^42^ in circulatory tumor cells and/or tumor initiating cells; and with EGFR^43^ and IGF1R^44^ to confer anticancer drug resistance. In addition, we have recently reported that TM4SF5 can bind to amino acid transporters such as the cystine/glutamate antiporter xc^−^ system, consisting of CD98hc and xCT (SLC7A11)^28^, and arginine transporters on lysosome and plasma membranes, depending on arginine sufficiency^45^, as described below in more detail. Thus, as a tetraspanin membrane protein, TM4SF5 can form protein complexes with amino acid transporters during pathological cell developments.

### TM4SF5 regulates ROS levels in lung epithelial cells

TM4SF5 binds to the cystine/glutamate transporter, the xc^−^ system consisting of CD98hc and xCT (SLC7A11) on the plasma membrane, especially in an environment with ROS in lung epithelial cells during lung fibrosis^28^.

#### The xc^−^ system for ROS modulation

Proper hormetic modulation of intracellular ROS levels is important for cellular functions and survival. ROS can be generated by mitochondrial respiration and nitric oxide synthase or by the action of multimeric NADPH oxidase complex following external cues such as exposure to radiation and anticancer drugs^46^. Intracellular ROS levels that are too high or low can affect the homeostatic functions of a cell^47^. Cells have antioxidant systems to attenuate cellular damage caused by ROS, involving antioxidant enzymes and small molecule antioxidants. Glutathione (GSH) has a nonprotein thiol that defends against intracellular ROS and GSH levels in all mammalian tissues. It is present at approximately 1–10 mM and is especially high in liver tissues (~5–10 mM)^48^. GSH is synthesized from precursor amino acids in an ATP-dependent two-step process catalyzed by two enzymes, gamma-glutamyl cysteine synthetase (γ-GCS) and GSH synthetase (GS)^49^; GCS forms peptide bonds between glutamate and cysteine, and glycine is then added to the compound by GS.

Recent studies have revealed that intracellular GSH levels can be used as important markers for several diseases^50,51^. Improper regulation of GSH level has thus been associated with many diseases, including HIV^52^, aging^53^, uremia^54^, pulmonary fibrosis^55^, and cancer^56,57^. Approximately 10–15% of intracellular GSH is located within mitochondria^58^, and mitochondrial malfunctions can lead to Parkinson’s disease^59^, Alzheimer’s disease^60^, and type 2 diabetes mellitus^61^. The mutation or deletion of genes involved in GSH synthesis also induces disease, and the activities of GCS and GS and the levels of GSH are decreased in Alzheimer’s disease^62^. Expression of the GCS enzyme promotes insulin release to attenuate hyperglycemia in diabetes mellitus patients^63^. Using an in vitro assay, intracellular GSH is depleted by treating NS20Y neuronal cell line with L-buthionine-(S,R)-sulfoximine to induce mitochondrial malfunction and apoptosis^59^. In contrast, the GSH level is higher in tumors in several types of cancers, including breast, ovarian, head and neck, and lung cancers, and lower in liver and brain cancers than in peripheral nontumor tissues^64^.

In addition to the proper regulation of gene expression for GSH synthesis, maintaining intracellular precursor amino acid levels is important for GSH synthesis. Most importantly, the concentration of intracellular cysteine is critical for glutathione synthesis, and the cellular cysteine importer is therefore important for ROS resistance and cell survival. The xc^−^ system is a dominant transporter for intracellular cysteine influx via exchanges of cystine (cysteine dimer) and glutamate in the plasma membrane^65,66^. The xc^−^ heterodimeric amino acid transporter is sodium-independent and has a high affinity for cystine and glutamate, leading to increased intracellular cystine and decreased glutamate concentrations^67^. Cystine is reduced to cysteine in the cytoplasm and used for the synthesis of glutathione. The xc^−^ transporter has two components: a heavy chain, 4F2 (SLC3A2, CD98hc), and a light chain, xCT (SLC7A11)^68^. SLC3A2 binds covalently with several amino acid transporters to form a heterodimeric transporter complex in the plasma membrane. In addition, LAT1 (SLC7A5) and LAT2 (SLC7A8) are well-known light chain transporters. These large neutral amino acid transporters import leucine, isoleucine and arginine by exporting glutamine^33^. Genomic disruption of xCT in pancreatic ductal adenocarcinoma cell lines decreases the import of cystine and cell survival when challenged by ROS-generating drugs^69^. In contrast, overexpression of xCT increases xc^−^ system activity and GSH synthesis in astrocytes^70^. Thus, the xCT light chain is important for cystine uptake and intracellular ROS resistance.

#### TM4SF5-mediated regulation of the xc^−^ system

We have recently reported that TM4SF5 functions as a novel regulator of the xc^−^ system^28^. The xCT light chain of the xc^−^ system in gastric cancer cells is regulated for stability in the plasma membrane by the CD44v8-10 variant of CD44, leading to intracellular ROS regulation^71^. In addition, in the case of lung epithelial cells in an inflammatory ROS-producing environment, CD98hc (SLC3A2), but not xCT (SLC7A11), is recruited to (and thereby stabilized at) the plasma membrane by TM4SF5, indicating that specific cell types have a unique signaling context. TM4SF5 specifically induces the CD44v8-10 variant via ZEB2 and epithelial splicing regulatory protein (ESRP) transcription regulators. Furthermore, the complex formation of TM4SF5 with the xc^−^ system is disrupted by a point mutation in CD44v8-10 at a residue that does not exist in the CD44 standard form (CD44s)^28^. Thus, TM4SF5-induced CD44v8-10, but not CD44s, can form a complex with the xc^−^ system, presumably within T_5_ERMs, leading to a TM4SF5-mediated increase in cystine uptake and cellular antioxidant GSH levels for hormetic ROS modulation during idiopathic pulmonary lung fibrosis (IPF).

#### TM4SF5-dependent ROS hormesis in type II alveolar epithelial cells (AECII) for IPF

In addition to the evidence from in vitro cellular systems, TM4SF5-mediated ROS regulation has been shown in an in vivo mouse model. Intratracheal injection of bleomycin into wild-type C57BL/6 mice induces severe pulmonary fibrosis to generate an experimental model for IPF, whereas administration of bleomycin into *Tm4sf5*-knockout mice shows reduced progression of fibrotic phenotypes and increased survival of mice^28^.

Interrupted reprogramming and the composition of alveolar cells can induce many diseases. In adult lungs, the alveolar epithelium consists of two major cell types, type I AECs (alveolar epithelial cells) and type II AECs, which occupy approximately 96 and 4% of the lung epithelium, respectively^72^. Type I AECs constitute thin layers of the lung epithelium for effective gas exchange, and type II AECs are cuboidal cells located in the alveolar corners^72^. Instead of providing a gas-exchanging surface, type II AECs are critical for injury repair and the homeostasis of alveolar cells by producing surfactants. Type II AECs can also function as facultative stem cells in the alveolar epithelium, leading to regeneration of type I AECs following epithelial injury^73^. Diverse studies have focused on the ablation of type II AEC and the clinical outcomes to demonstrate the critical role of type II AEC in maintaining lung homeostasis and in new therapies for several lung diseases. Type II AEC depletion alters lung morphology, pulmonary function, collagen deposition, (apoptosis-related) protein expression, and tissue repair and remodeling^74^. During IPF development, type I AECs undergo apoptosis, and type II AECs are hyperplasic and hypertrophic^75^. Many features of type I AECs and type II AECs are unique to disease development, with functions that involve cell fate and protein expression. Molecular analysis of the lung tissues from the bleomycin-treated C57BL/6 mice revealed that the expression of Tm4sf5 is lower in primary type I AECs than in type II AECs. In primary type II AECs, Tm4sf5 mediates induction of a CD44v8-10 splicing variant by repressing ZEB2 to elevate ESRP1 transcription^28^, which leads to Tm4sf5-dependent ROS resistance by active cystine uptake for intracellular antioxidant GSH synthesis. As a result, *Tm4sf5*-knockout C57BL/6 mice have ameliorated bleomycin-induced lung fibrosis compared to the fibrosis in *Tm4sf5*-expressing wild type mice, in which the architecture of the lung epithelium is disrupted, leading to the accelerated death of type I AECs and hyperplasia of type II AECs. TM4SF5 and CD44v80-10 are therefore promising targets for the treatment of pulmonary fibrosis.

### The lysosomal arginine transporter SLC38A9, mTORC1, and TM4SF5

#### mTORC1 and amino acids

Mechanistic target of rapamycin complex 1 (mTORC1) is a protein complex composed of mTOR, raptor, mammalian lethal with SEC13 protein, protein-rich AKT1 substrate 1, and DEP domain-containing mTOR-interacting protein. mTORC1 is regulated by various external cues, such as those transduced by insulin, growth factors, glucose and amino acids, to control protein synthesis^76^. Activated mTORC1 is recruited to the surface of lysosomal membranes to interact with various machinery proteins to promote protein translation, lipid synthesis, and nucleotide synthesis and to inhibit autophagy/lysosome biogenesis^77^. Recently, scientists have focused on identifying the mechanisms by which amino acids activate mTORC1. The complicated mechanism through which Rag GTPase switches mTORC1 on or off depends on amino acid sufficiency^78^. Based on Rag GTPase, numerous proteins binding to mTORC1 comprise an amino acid activating machinery complex on the lysosome surface. Importantly, leucine and arginine in the liver are key amino acids that activate mTORC1, and extensive studies have focused on identifying the physiological sensors of lysosomal leucine and arginine.

#### SLC38A9 and mTORC1

Advances in proteomics analysis have enabled scientists to identify SLC38A9, which is the transporter at the lysosomal membrane that transports arginine from the lumen to the cytosol. SLC38A9-mediated arginine transport leads to activation of mTORC1 signaling^79,80^. Although the function of SLC38A9 has not been clarified, according to the SLC table at Bioparadigm.org, SLC38A9 belongs to the sodium-coupled neutral amino acid transporter family; Wyant et al. reported that SLC38A9 is an arginine sensor that transports several amino acids out of the lysosomal membrane to activate mTORC1^81^. The identification of SLC38A9 as a factor in the arginine-sensing machinery has significantly contributed to our understanding of the mechanism through which arginine activates mTORC1 signaling. In addition, a cytoplasmic arginine sensor, Castor1, regulates GATOR to turn Rag GTPase on and off^82^. However, SLC38A9 is found to weakly bind arginine in lysosomes at micromolar levels^81^; therefore, another physiological arginine sensor has been expected to exist.

#### Arginine transport

Arginine is a crucial component of cell survival because it is a precursor of many building blocks of cellular processes. Arginine functions as an amino acid in protein synthesis and has multiple metabolic fates that transform it into nitric oxide, creatine, ornithine, citrulline, polyamines, agmatine, and urea^83^. Arginine is transported from the extracellular space to the cytoplasm by arginine transporters in the plasma membrane, such as SLC7A1, 2, and 3. Because arginine depletion can jeopardize cellular functions, arginine can be resynthesized in cells using the urea cycle when imported arginine is lacking^83^. Many types of cancer cells, especially those of hepatocellular carcinoma (HCC), kidney carcinoma, malignant melanoma, and prostate carcinoma, are deficient in argininosuccinate synthase 1 (ASS1), which is a key enzyme that regenerates arginine^84^. Therefore, ASS1-deficient HCC cells primarily rely on external arginine importation or even lysosomal arginine generated from protein degradation. These characteristics of arginine auxotrophs of HCC have led to the development of arginine-deprivation therapy (ADT), which is used to deplete external arginine through recombinant enzymes, such as arginine deaminase-PEG and rhArginase-PEG^83^. ADT was promising in the early phases, but the outcomes were not successful due to re-expression (or activation) of ASS1, leading to resistance. However, targeting arginine in cancer is still an attractive approach because it affects only cancer cells. It is also reasonable to assume that survival and proliferation of HCC cells can be suppressed by blocking or inactivating the arginine transporters.

#### TM4SF5 in arginine transport

We recently reported that TM4SF5 can translocate from the plasma membrane to the lysosomal membranes, depending on extracellular (lysosome lumen after arginine is transported) arginine at physiological levels^45^. Depletion of amino acids or arginine causes increased TM4SF5 localization to the plasma membrane, but repletion of them leads to greater TM4SF5 localizing to the membrane of lysosomes. Although arginine sufficiency leads to mTOR translocation to lysosomes, TM4SF5 expression itself can also cause the translocation of mTOR to lysosomes. Therefore, a complex with TM4SF5 and the SLC7A1 arginine transporter in the plasma membrane is formed as an alternative to the TM4SF5 complex formed with the lysosomal arginine transporter SLC38A9. Under conditions of amino acid deprivation, particularly arginine deprivation, TM4SF5 binds to SLC7A1 following translocation to the plasma membrane, whereas TM4SF5 translocates to lysosomal membranes upon amino acid or arginine repletion, leading to complex formation with SLC38A9 and mTOR there. Furthermore, TM4SF5 can directly bind free L-arginine via hydrogen bonds with specific residues within the long extracellular loop of TM4SF5 (^124^WGYHFE^129^). SLC38A9 itself has been shown to be too weak (with Km values at mM levels) to bind L-arginine^80^ in the lysosomal lumen of HEK293 cells at 50–250 μM^81^. Direct binding of TM4SF5 to L-arginine with an EC_50_ of 10.48–37.9 μM suggests that TM4SF5 is a physiological sensor of lysosomal arginine in liver cancer cells. The direct binding of arginine to TM4SF5 can be beneficial for the SLC38A9-dependent efflux of L-arginine, which is locally sequestered via TM4SF5 binding^45^. Arginine efflux from the lysosomal lumen to the cytosol can activate a component of mTORC1, S6K1. Thus, the TM4SF5-mediated protein complex (i.e., T_5_ERMs on the lysosomal membrane) together with mTORC1 can activate S6K1, leading to protein translation and cell proliferation. Moreover, TCGA data show that TM4SF5 alone or with mTOR is significantly related to poor outcomes for recurrence-free survival, although mTOR itself is not a significant factor for this poor outcome. The use of anti-TM4SF5 reagents such as TSAHC can be a strategy to impair arginine auxotrophs in HCC. Blockading amino acid transporters can thus be more beneficial than deprivation or removal of the amino acids around liver cancer cells.

## Conclusion

The function of TM4SF5, as a member of the tetraspanin, has been intensively studied regarding its effects on liver pathology. Notably, although its physiological ligand is unknown, TM4SF5 interacts with diverse membrane receptors to transduce signaling to regulate cellular functions. Thus, its association with (an)other membrane protein(s) on cellular membranes may be considered as a ligand-binding process because it can lead to stabilization, trafficking, and activation of binding partners. Addressing different membrane proteins and receptors, we have discussed the evidence suggesting that two different amino acid transporter systems bind to TM4SF5, the cystine/glutamate antiporter (xc^−^ system with CD98hc and xCT) on lung epithelial cells and the arginine transporter (SLC7A1 on the plasma membrane and SLC38A9 on the lysosomal membrane) of hepatocytes. Although more evidence on the roles of TM4SF5 in amino acid transporters during homeostatic and pathological conditions needs to be obtained, T_5_ERMs, including transporters, can play important roles in modulating cellular functions in a spatiotemporal manner. Because amino acid transporters are involved in the homeostatic balancing of extracellular and intracellular amino acids, their malfunction could easily lead to metabolic disorders, which may be complicated by immunological environments. The roles of TM4SF5 and the physiological significance of T_5_ERMs in influencing amino acid availability inside and outside of cells should therefore be further characterized in the context of different immune-related metabolic disorders.
